# Iron status, inflammation and hepcidin in ESRD patients: The confounding role of intravenous iron therapy

**DOI:** 10.4103/0971-4065.70840

**Published:** 2010-07

**Authors:** A. Jairam, R. Das, P. K. Aggarwal, H. S. Kohli, K. L. Gupta, V. Sakhuja, V. Jha

**Affiliations:** Department of Nephrology, Postgraduate Institute of Medical Education and Research, Chandigarh, India; 1 Department of Hematology, Postgraduate Institute of Medical Education and Research, Chandigarh, India

**Keywords:** Anemia, end-stage renal disease, hepcidin, inflammation, intravenous iron

## Abstract

Uremia is a state of heightened inflammatory activation. This might have an impact on several parameters including anemia management. Inflammation interferes with iron utilization in chronic kidney disease through hepcidin. We studied the body iron stores, degree of inflammatory activation, and pro-hepcidin levels in newly diagnosed patients with end-stage renal disease (ESRD), and compared them with normal population. In addition to clinical examination and anthropometry, the levels of iron, ferritin, C-reactive protein, tumor necrosis factor alfa, interleukin-6, and prohepcidin were estimated. A total of 74 ESRD patients and 52 healthy controls were studied. The ESRD patients had a significantly lower estimated body fat percentage, muscle mass, and albumin; and higher transferrin saturation (TSAT) and raised serum ferritin. Inflammatory activation was evident in the ESRD group as shown by the significantly higher CRP, IL-6, and TNF-α levels. The pro-hepcidin levels were also increased in this group. Half of the ESRD patients had received parenteral iron before referral. Patients who had received intravenous iron showed higher iron, ferritin, and TSAT levels. These patients also showed more marked inflammatory activation, as shown by the significantly higher CRP, TNF-α, and IL-6 levels. We conclude that our ESRD patients showed marked inflammatory activation, which was more pronounced in patients who had received IV iron. High hepcidin levels could explain the functional iron deficiency. The cause of the relatively greater degree of inflammatory activation as well as the relationship with IV iron administration needs further studies.

## Introduction

Anemia contributes significantly to the morbidity and mortality in chronic kidney diseases (CKD). The chief etiology of anemia in CKD is erythropoietin (Epo) deficiency. Despite the widespread Epo use, over 50% of the patients do not reach the target hemoglobin levels.[1,2] The most common reason for poor response to Epo therapy is iron deficiency.[1] Inflammation has been implicated as another important cause of poor response.[3] C-reactive protein (CRP), interleukin-6 (IL-6), and tumor necrosis factor alpha (TNF-α) are acute phase reactants that have been used to reliably assess the degree of inflammatory activation.[4] Hepcidin, a regulator of body iron stores, has been recently discovered to play a critical role in the pathogenesis of anemia of chronic disease.

Ferritin, other than being a marker of body iron stores, also increases in acute inflammation, and becomes less valuable as an indicator of iron status during inflammation.[5] Parenteral iron therapy has been shown to improve body iron stores and reduce Epo requirement. However, studies have suggested that parenteral iron therapy might itself contribute to morbidity and mortality by inducing a pro-inflammatory state, due to increased oxidative stress.[6] Additionally, assessment of iron status itself may be rendered difficult on account of inflammatory activation.

Indian subjects have been demonstrated to have microinflammation and higher body fat percentage in healthy subjects as compared to Caucasians.[7,8] A vast majority of Indians are vegetarians, and anemia due to iron deficiency is very common in the general population.[9] Additionally, due to the logistic and financial constraints, management of ESRD is usually suboptimal. In this scenario, the inflammatory activation, iron profile, and anthropometry are likely to very different in Indian CKD patients. To date, these issues have not been addressed in Indian CKD patients. In this study, we evaluated the body iron stores, degree of inflammatory activation, and hepcidin levels in newly diagnosed patients with end-stage renal disease, and compared them with the normal population. Furthermore, we investigated if parenteral iron therapy had any effect on the status of inflammation.

## Materials and Methods

This cross-sectional study was conducted at the Postgraduate Institute of Medical Education and Research, a large tertiary care referral hospital in north India. The study subjects were newly diagnosed ESRD patients of either sex. The exclusion criteria were: age less than 18 years, evidence of acute infection or trauma in the last four weeks, history of parenteral iron injection in the last 14 days, history of blood transfusion in the last one month, hemoglobinopathies, malignancy, recent overt blood loss, and post-transplant status. Healthy adult individuals were recruited as controls. To ensure homogeneity between the control and ESRD population, healthy individuals were selected from the friends and relatives accompanying the ESRD patients. General physical examination, urinalysis and blood sugar, and creatinine estimation were done to establish the healthy nature of controls. Informed consent was obtained from all subjects. A total of 74 patients and 52 controls were studied.

All patients underwent a thorough physical examination. In order to assess the nutritional status, the following anthropometrical data were determined for all patients: height (without footwear), weight, body mass index, mid-arm circumference (MAC), and waist-to-hip ratio. Skin fold thickness was measured at the triceps, biceps, and the subscapular and suprailiac regions using modified skin callipers. Mid arm muscle circumference (MAMC) was calculated from the formula: MAMC = MAC - *π* times the triceps skin fold thickness. Body fat percentage was calculated by the Durnin Womersley method, from the logarithm of the sum of the four skin fold thicknesses.[10] Body mass index was calculated as follows: BMI = Weight (kg)/[Height (m)]^2^. For dialysis patients, the modality and schedule of dialysis were also recorded. Hemogram, serum iron, total iron binding capacity (TIBC), serum ferritin, percentage transferrin saturation (TSAT), TNF-α, IL-6, and quantitative CRP levels were studied. If a patient was on oral iron, it was stopped for a week before sampling. Fasting venous blood samples were collected in chilled tubes; the serum was separated and frozen at –80° C for subsequent estimation of C-reactive protein, ferritin, IL-6, TNF-alpha, and pro-hepcidin. All the samples were run in duplicate. The methodology used for estimating was as follows:

*Serum iron:* Serum iron was measured as recommended by the International Committee for Standardization in Hematology. Unknown samples were tested along with a blank and the standard. Protein was precipitated, and a chromogen was added to the supernatant followed by measurement of the absorbance. The serum iron level was given by the following equation:

$$\mathrm{Serum}\;\mathrm{iron}\;=\;\frac{A_{562}\;\mathrm{test}\;-\;A_{562}\;\mathrm{blank}}{A_{562}\;\mathrm{standard}\;-\;A_{562}\;\mathrm{blank}}\;\times \;100$$

*Total iron binding capacity:* Excess iron was added to the sample as ferric chloride. Excess unbound iron was removed with magnesium carbonate. The iron concentration was measured using the method given earlier in the text.

*Serum ferritin:* Ferritin is estimated by an immunometric enzyme immunoassay (ORGenTec, Mainz, Germany). The assay is based on microplates coated with specific anti-human ferritin antibodies. The binding of the analyte as well as the formation of the sandwich complex and enzymatic color reaction takes place during three different reaction phases, and the intensity of the developed color is proportional to the concentration of the ferritin present in the sample.

C-reactive protein was estimated using a quantitative CRP assay as well as a highly sensitive C-reactive protein assay kit, according to the manufacturer’s protocol. The test samples were initially estimated by the quantitative CRP assay that had a range of 8 – 160 mg/l. The samples that gave readings less than 10 mg/l were subjected to the highly sensitive CRP assay (range 0.005 – 16 mg/l). Thus, both assays together covered the entire range of CRP levels. The controls were initially subjected to the highly sensitive assay and if they had a value greater than 10 mg/l, the assay was repeated using the quantitative standard CRP assay. The Orion Diagnostic CRP assay (Cat No.67274) was used for the quantitative CRP assay. The principle of the test was based on the measurement of immunoprecipitation in a liquid phase. Antibodies against human CRP were added to an aliquot of patient serum and CRP buffer. The antibodies underwent an agglutination reaction with CRP in the serum, resulting in an increase in the turbidity of the mixture. The turbidity was measured using the Clima plus–RA 133000 clinical chemistry analyzer at a wavelength of 340 nm. The Sentinel Ch. CRP Ultra assay (Milano, Italy) was used for the highly sensitive CRP assay. The anti-CRP antibody adsorbed to the latex particles was added to the serum sample, the resulting agglutination was measured using the Clima plus–RA 133000 clinical chemistry analyzer at a wavelength of 550 – 580 nm. Intra-individual variability was looked for by running the Standard and Ultra CRP assays twice, for five samples. The IL-6 levels were assayed using the Immunotech IL-6 enzyme immunoassay. The ELISA was a one-immnunological step sandwich type assay. The samples and standards were incubated in the microtiter plate coated with the first monoclonal antibody anti-IL-6, in the presence of the second anti-IL-6 monoclonal antibody linked to an acetylcholinesterase. After incubation, the wells were washed and the bound enzymatic activity was detected by the addition of a chromogenic substrate. The intensity of the coloration was proportional to the IL-6 concentration in the sample.

TNF-α in the serum was assayed using the Immunotech TNF-α enzyme immunoassay. The ELISA was a one-step immunological step sandwich type assay. The samples and standards were incubated in the microtiter plate coated with the first monoclonal antibody anti-TNF-α, in the presence of the second anti-TNF-α monoclonal antibody linked to an alkaline phosphatase. After incubation, the wells were washed and the bound enzymatic activity was detected by addition of a chromogenic substrate. The intensity of the coloration was proportional to the TNF-α concentration.

Pro-hepcidin was estimated using the DRG Hepcidin pro-hormone enzyme immunoassay kit. The test was a solid phase enzyme-linked immunosorbent assay (ELISA), based on the principle of competitive binding. The test sample was incubated in microtiter wells coated with polyclonal antibody directed towards an antigenic site on hepcidin pro-hormone. The hepcidin pro-hormone-biotin conjugate added, competed with the endogenous hepcidin pro-hormone of the test sample. The amount of bound biotin conjugate was inversely proportional to the concentration of the hepcidin pro-hormone. After the substrate solution was added, the intensity of the color developed was inversely proportional to the concentration to the hepcidin pro-hormone.

Data were presented as mean ± S.E. Continuous data was compared using Student’s T test for the normally distributed and Mann Whitney’s U test for the skewed data. Categorical variables were analyzed using the Chi-square test. Bivariate correlation was carried out between the anthropometric parameters, iron status, and inflammatory markers to determine the strength of the association using Spearman’s coefficient.

## Results

A total of 126 adults were studied: 74 ESRD patients and 52 healthy controls. The baseline data of the subjects are shown in Table 1. There was no difference in the gender distribution or height between the cases and controls. However, the patients were younger and weighed less. The average duration of the kidney disease was 10.2 months.

**Table 1:** Baseline characteristics of the study population

Parameter	ESRD n = 74	Controls n = 52	*P* value
Age (years)	36 ± 11.9	40.4 ± 9.2	<0.05
Sex (Male/female)	54/20	33/19	>0.05
Height (cm)	166.8 ± 7.1	166.8 ± 8.1	>0.05
Weight (kg)	53.9 ± 8.04	68.1 ± 8.04	<0.01
BMI (kg/m^2^)	19.4 ± 3.04	24.5 ± 2.06	<0.01
On dialysis	49%	-	
HD	41%	-	
CAPD	8%	-	

HD – hemodialysis, CAPD – continuous ambulatory peritoneal dialysis

**Figure 1:**
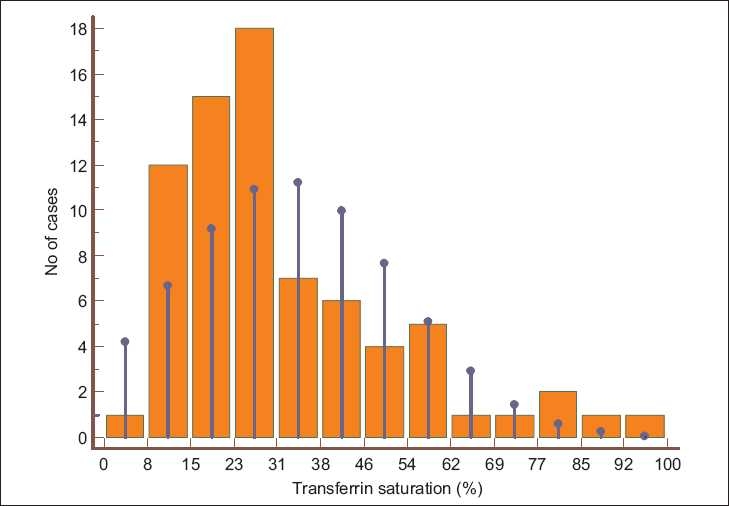
Histogram showing transferrin saturation in ESRD patients. The vertical blue lines indicate normal distribution

**Figure 2:**
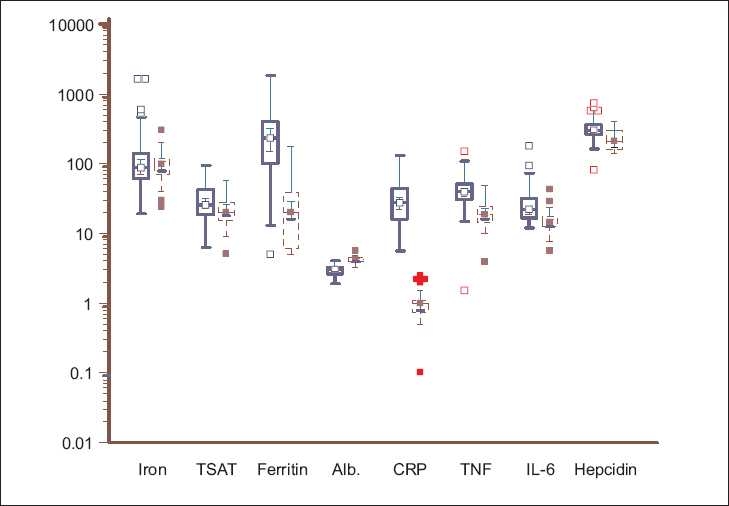
Box-and-whisker plots showing the serum iron (microgm/dl), transferrin saturation (TSAT, %), ferritin (ng/ml), albumin (g/dl), C-reactive protein (mg/l), TNF-a (pg/ml) IL-6 (pg/ml), and hepcidin (ng/ml) in ESRD patients (solid blue line) and healthy controls (dashed red line). Log-transformed data, with the solid horizontal line denoting the median along with the interquartile range

The anthropometric parameters, iron profile, and inflammatory markers of the two groups are shown in Table 2 and Figure 1. The ESRD patients had a significantly lower estimated body fat percentage and muscle mass, higher TSAT, and markedly raised serum ferritin levels. In the control population, iron deficiency was common (51%), whereas, iron overload was very rare (4%). Figure 2 shows the TSAT distribution among the ESRD subjects: about 20% had TSAT > 50% and 40% had TSAT< 25%. Half of the ESRD patients had received parenteral iron. Examination of the referral records showed that 47% had received an iron sucrose preparation, whereas, in the rest, the formulations were unknown or other forms of iron had been administered. Twenty-nine percent had received blood transfusions in the past (mean 2.2 units). Only 32 patients had received Epo, with most getting only a few doses or they took it intermittently.

**Table 2:** Anthropometric parameters, iron status and inflammatory markers in ESRD and control population

Characteristic	ESRD n = 74	Controls n = 52	*P* value
Waist:Hip ratio	0.88 ± 0.11	0.95 ± 0.04	<0.001
Σ skin fold thickness (mm)	24 ± 10.1	60.4 ± 25.6	<0.0001
Body fat percent (%)	12.8 ± 6.1	27.0 ± 5.8	<0.0001
MAMC (cm)	22.7 ± 3.3	25.2 ± 3.2	<0.0001
Serum albumin (g/dl)	2.96 ± 0.06	4.16 ± 0.06	<0.0001
Hemoglobin (g/dl)	8.1 ± 0.2	13.8 ± 0.2	<0.0001
Iron (microgm/dl)	153.4 ± 31.6	100.3 ± 6.7	0.78
TSAT (percent)	32.2 ± 2.3	22.5 ± 1.5	0.007
Serum ferritin (ng/ml)	331.7 ± 39.56	28.3 ± 4.1	<0.001
Serum CRP (mg/l)	35.8 ± 3.4	1.01 ± 0.06	<0.00
Interleukin-6 (pg/ml)	29.6 ± 3.04	15.7 ± 1.05	<0.001
TNF-α (pg/ml)	43.1 ± 2.7	20.7 ± 1.4	<0.001
Hepcidin (ng/ml)	332.2 ± 17.5	241.9 ± 18.4	0.01

(BMI-Body mass index, Σ Skin fold thickness – sum of the triceps, biceps, subscapular, and suprailiac skin fold thicknesses, MAMC – mid-arm muscle circumference, TSAT- Transferrin saturation, CRP – C reactive protein, TNF-α - Tumor necrosis factor alpha)

Evidence of marked inflammatory activation was demonstrated in the ESRD group, as was seen by significantly higher CRP, IL-6, and TNF-α levels. The pro-hepcidin levels were also increased in this group. Six (12%) of the controls were obese using the WHO standards of BMI > 28.6 kg/m^2^ for females and BMI > 30 kg/m^2^ for males.[11] The CRP level in these subjects was 1.35±0.16 mg/l, significantly higher than the rest of controls 0.97±0.09 mg/l (*P* = 0.045).

In the ESRD group, there was a significant inverse correlation of the CRP levels with the body fat percentage (*P* = 0.009). The ferritin levels correlated significantly with the TSAT (rho = 0.443, *P* = 0.0002), but not with CRP (rho = 0.09, *P* = 0.44). However, a correlation between ferritin and CRP could be shown when values greater than 500 ng/ml were excluded (rho = 0.32, *P* = 0.045). CRP values correlated with TNF-α (rho = 0.335, *P* = 0.006), and IL-6 (rho = 0.372, *P* = 0.002) and showed a negative correlation with serum albumin (rho = - 0.487, *P* <0.0001). There was a good correlation between IL-6 and TNF-α levels (rho = 0.459, *P* =0.0002). The pro-hepcidin level showed a trend to vary with the transferrin saturation (rho = 0.219, *P* = 0.09), but there was no correlation with the serum iron or ferritin levels.

**Figure 3:**
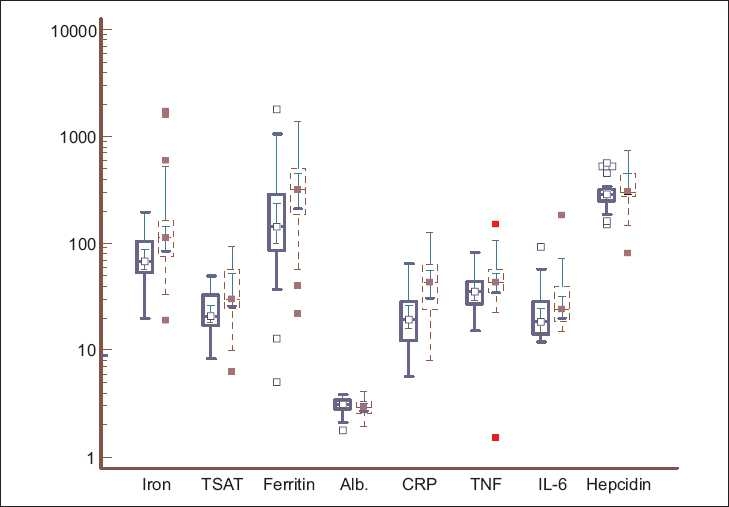
Box-and-whisker plots showing the serum iron (microgm/dl), transferrin saturation (TSAT, %), ferritin (ng/ml), albumin (g/dl), C-reactive protein (mg/l), TNF-a (pg/ml), IL-6 (pg/ml), and hepcidin (ng/ml) in ESRD patients who did not receive IV iron (solid blue line) and those who did (dashed red line). Log-transformed data, with the solid horizontal line denoting the median along with the interquartile range

The patients were divided into two groups on the basis of a history of their receiving parenteral iron. As expected, patients treated with iron showed higher iron, ferritin, and TSAT levels. These patients also showed evidence of inflammatory activation, as shown by significantly higher CRP, TNF-α, and IL-6 levels [Table 3 and Figure 3].

**Table 3:** Iron status and inflammatory markers in patients who did or did not receive IV iron before referral

Characteristic	No iron n = 37	Received IV iron n = 37	*P* value
Serum albumin (g/dl)	2.96 ± 0.06	4.16 ± 0.06	<0.0001
Iron (microgm/dl)	81.6 ± .67	225.3 ± 60.4	0.002
TSAT (percent)	24.1 ± 1.8	39.9 ± 3.9	0.0023
Serum ferritin (ng/ml)	285.9 ± 57.6	397.5 ± 52.8	<0.0076
Serum CRP (mg/l)	22.15 ± 2.05	49.5 ± 5.7	<0.0001
Interleukin-6 (pg/ml)	23 ± 2.8	35.4 ± 5.3	<0.0081
TNF-α (pg/ml)	37.3 ± 2.6	48.9 ± 4.6	<0.001
Prohepcidin (ng/ml)	307.7 ± 19.8	359.3 ± 52.8	0.12

BMI-Body mass index, Σ Skin fold thickness – sum of the triceps, biceps, subscapular, and suprailiac skin fold thicknesses, MAMC – mid arm muscle circumference, TSAT- Transferrin saturation, CRP – C reactive protein, TNF-α - Tumor necrosis factor alpha

## Discussion

Anemia is an important contributor to morbidity and mortality in ESRD. Anemia management was revolutionized after the advent of recombinant human erythropoietin and later parenteral iron therapy. However, despite their widespread use, the modest target of hemoglobin 11 to 12 g/dL has been difficult to achieve.[12]

Iron deficiency is rampant in the general Indian population, with reported prevalence of nutritional anemia being 33 – 98%.[13,14] The cause is thought to be due to poor nutrition stemming from a predominantly vegetarian diet. Indian studies on anemia in CKD also identified iron deficiency as a major problem.[15,16] Surprisingly, about 60% of the ESRD patients in this study had adequate iron stores (TSAT > 25%), most of whom had received parenteral iron therapy before presentation. Deficiency was common (62%), however, among those who had not received IV iron. Due to a combined effect of the wide dissemination of guidelines advocating parenteral iron and availability of relatively safe parenteral iron formulations, they are being increasingly used by the referring physicians. Hence, evaluation in tertiary care hospitals like ours is likely to show a skewed picture. In another study from a referral hospital, 75% patients showed evidence of iron overload during pre-transplant evaluation.[17]

The major finding of the study was the demonstration of near universal inflammatory activation in the study population. The CRP levels were increased over 40-fold; and TNF-α and IL-6 levels were significantly raised as compared to those of the controls (Z score was > 2 in 64%, and 44% for TNF-α and IL-6, respectively). The proposed causes included activation of immune cells (monocytes and T cells); reduced antioxidant activity; reduced clearance, and coexistent subclinical infections or autoimmune diseases. In patients on dialysis, exposure to dialysis membranes in hemodialysis patients, and bioincompatible fluids in CAPD patients also contributed.[18] Intercurrent infections worsened the situation. Only a percentage of the patients in this study had received dialysis, and those with recent infection had been excluded. As this is the first study to evaluate the status of inflammation in Indian CKD patients, it is possible that some unique factors related to the ethnicity such as the genetic background or dietary habits contributed to this status. It has been shown that compared to Caucasians, even ‘healthy’ Indians have elevated CRP levels.[7,19] In a study involving 377 healthy Indian adults, the CRP levels correlated with the body fat.[20] The CRP levels in the controls did not correlate with the waist : hip ratio, BMI or body fat percentage, probably due to small numbers. Among the obese control subjects, the level was significantly higher, suggesting that Indian subjects tend to have micro-inflammation. The cause is unclear at the present time, but could be at least partly associated with increased body fat. It is tantalizing to propose that this tendency to microinflammation gets exaggerated with the onset of CKD.

Inflammation has been implicated in several complications in CKD, including malnutrition and accelerated atherosclerosis. It also blunts the iron utilization and induces resistance to erythropoietin therapy. The exact pathway by which the inflammatory cascade results in erythropoietin resistance is not completely understood. It has been hypothesized that inflammatory activators have a pro-apoptotic effect on erythroid progenitor cells and compete with the anti-apoptotic effect of erythropoietin, the end result being erythropoietin resistance.[21] Pentoxyfylline, a nonspecific anti-TNF-α antagonist, has shown encouraging results in erythropoietin unresponsive CKD patients.[22]

The anthropometric measurements confirmed the high prevalence of malnutrition in our ESRD cohort. Close to 90% of the malnourished patients had evidence of inflammatory activation. There was a strong inverse correlation between serum albumin and CRP. The link between inflammation and malnutrition was well-established, and our findings suggested that measures to combat inflammation would be needed to improve the nutritional status of these patients.

We also found elevated levels of pro-hepcidin, a precursor of hepcidin, among ESRD patients. Hepcidin, a defensin-like circulatory peptide was the major iron-regulatory hormone that linked innate immunity and iron metabolism. It was isolated as a small 25 amino acid cysteine-rich peptide from human plasma and urine, and was seen to have antimicrobial activity.[23,24] It was produced by the liver in response to inflammatory stimuli. It downregulated ferroportin, thereby decreasing iron release from the enterocytes and macrophages and causing secondary hypoferremia.[25] This property was useful for protection against the invading pathogens, although it reduced iron availability for erythropoiesis. Hepcidin also inhibited the proliferation and survival of erythropoietin progenitor cells.

The interaction of pro-inflammatory cytokines with hepcidin in the genesis of functional iron deficiency in CKD patients is an area of intense research. Several studies have shown elevated hepcidin levels in CKD, and it is now considered to be the critical link between inflammation and anemia in CKD patients.[26] Epo hyporesponsive patients exhibit a more marked inflammatory activation and higher hepcidin levels.[27] A recent study has shown alteration in the hepcidin gene expression by IL-6.[28] The hepcidin levels correlate weakly with TSAT, but not with any of the markers of inflammation. In the other studies too, hepcidin correlates with TSAT and ferritin, but correlation between hepcidin and inflammation is inconsistent.[29–32] The future of the management of erythropoietin resistance could revolve around countering inflammation and modulating the hepcidin effect.

A significant proportion of the ESRD patients exhibited iron overload (TSAT > 50%). In part, this could be due to unmonitored administration of parenteral iron, without evaluating the iron status, and repeated blood transfusions. Production of hepcidin by the inflammatory activators could cause disturbed release of iron from the reticuloendothelial system resulting in high ferritin levels and non-utilization of the parenteral iron administered.[33]

Use of ferritin levels to define iron overload may be faulty because of the two-to-three fold elevations of ferritin levels with inflammatory activation. However, in the present study an excellent correlation was noted between ferritin and TSAT levels. Kalantar-Zadeh *et al*. also noted a good correlation of serum ferritin with TSAT and not CRP.[5] This was counterintuitive, as ferritin was a well-known acute phase reactant. When the analysis was repeated after excluding patients with ferritin < 200 or > 2000 ng/ml, a significant correlation was noted. In the present study, when serum ferritin values > 500 ng/ml were excluded, a significant correlation between CRP and ferritin emerged. Our study confirmed the view that ferritin reflected iron levels at all ranges, but was influenced by inflammation at values below 500 ng/ml. In other words, if the serum ferritin was markedly increased, it was more likely to be a result of iron overload and not just due to inflammation.

Parenteral iron has emerged as an important tool in anemia management in CKD, either by itself or when combined with Epo.[34] However, our findings suggest that there could be a downside to an indiscriminate administration of IV iron, as it probably contributes to the inflammatory state. The levels of all the markers of inflammatory activation were increased in those who had received IV iron. A small fraction of the parenteral iron was redox-active. The ability of IV iron to increase the oxidative stress in dialysis patients was well-documented.[35–39] Malyszko *et al*.[40] showed an increase in hepcidin levels following IV iron therapy in dialysis patients, but did not measure other inflammatory markers. In experimental animals, the iron sucrose injection potentiated increased TNF-α production, following intraperitoneal endotoxin administration.[41] This was not seen with iron dextran or ferric gluconate. Our findings are contrary to those of Weiss *et al*.,[42] who showed a reduction in IL-6 levels in HD patients who received IV iron with Epo, but not in those who received Epo alone. Administration of IV iron beyond the point of iron repletion will increase the non-transferrin bound or labile plasma iron, which is more likely to cause oxidative stress.[43] As only a small fraction of our patients were on Epo, it is possible that continued administration of iron in the absence of utilization produced a quick saturation of transporter proteins and increase in active labile form. These findings are of potential clinical significance and need to be verified in appropriately designed trials. A majority of our patients received iron sucrose. We need to find out if this finding is also seen with other formulations. Whether newer preparations such as iron ferumoxytol will be superior in this respect needs to be studied. There is a need to find a formulation that corrects the iron deficiency without perturbing the inflammatory milieu.

There were several limitations to this study. First, the patient population was heterogenous, consisting of dialyzed and non-dialyzed ESRD patients, who had presented to a tertiary care center. Second, as this was a cross-sectional study, we could not document if the findings were persistent. Third, we were unable to document the exact nature of the IV iron formulation, the precise dose or the manner of administration. Fourth, we measured the hepcidin pro-hormone rather than the biologically active forms. Assays for the latter have now become available. Finally, this study only shows an association, and cannot prove the causality. Interventional studies will be needed to finally nail down a cause-and-effect relationship.

In conclusion, there was marked inflammatory activation in our ESRD patients. The activation was more pronounced in patients who had received IV iron. High hepcidin levels could explain the functional iron deficiency. The cause of the relatively greater degree of inflammatory activation as well as the relationship with IV iron administration needs further studies.

## References

[1] KDOQI; National Kidney Foudnation (2006). II. Clinical practice guidelines and clinical practice recommendations for anemia in chronic kidney disease in adults. Am J Kidney Dis.

[2] Collins AJ, Li S, Ebben J, Ma JZ, Manning W (2000). Hematocrit levels and associated medicare expenditures. Am J Kidney Dis.

[3] MacDougall IC (2001). Hyporesponsiveness to anemia therapy: What are we doing wrong?. Perit Dial Int.

[4] Kalantar-Zadeh K, Kopple JD, Humphreys MH, Block G (2004). Comparing outcome predictability of markers of malnutrition-inflammation complex syndrome in haemodialysis patients. Nephrol Dial Transplant.

[5] Kalantar-Zadeh K, Rodriguez RA, Humphreys MH (2004). Association between serum ferritin and measures of inflammation, nutrition and iron in haemodialysis patients. Nephrol Dial Transplant.

[6] Feldman HI, Joffe M, Robinson B, Knauss J, Cizman B, Guo W (2004). Administration of parenteral iron and mortality among hemodialysis patients. J Am Soc Nephrol.

[7] Chambers JC, Eda S, Bassett P, Karim Y, Thompson SG, Gallimore JR (2001). C-reactive protein, insulin resistance, central obesity, and coronary heart disease risk in Indian Asians from the United Kingdom compared with European whites. Circulation.

[8] Misra A (2004). C-reactive protein in young individuals: Problems and implications for Asian Indians. Nutrition.

[9] Mehta BC (1990). Iron deficiency. Prevalence and problems. J Assoc Physicians India.

[10] Durnin JV, Womersley J (1974). Body fat assessed from total body density and its estimation from skinfold thickness: Measurements on 481 men and women aged from 16 to 72 years. Br J Nutr.

[11] (1984). World Health organization: Techn Rep. Ser., No 702.

[12] Barany P (2001). Inflammation, serum C-reactive protein, and erythropoietin resistance. Nephrol Dial Transplant.

[13] (2002). (NFHS-II) NFHS-I: Anemia among women and children.

[14] Sheshadri S (1997). Nutritional anemia in South Asia.

[15] Aggarwal HK, Nand N, Singh S, Singh M, Hemant, Kaushik G (2003). Comparison of oral versus intravenous iron therapy in predialysis patients of chronic renal failure receiving recombinant human erythropoietin. J Assoc Physicians India.

[16] Gupta M, Kannan M, Gupta S, Saxena R (2003). Contribution of iron deficiency to anemia in chronic renal failure. Indian J Pathol Microbiol.

[17] John GT, Chandy M, Thomas PP, Shastry JC, Jacob CK (1993). Iron stores in patients on haemodialysis after renal transplantation. Natl Med J India.

[18] Kalantar-Zadeh K, Ikizler TA, Block G, Avram MM, Kopple JD (2003). Malnutrition-inflammation complex syndrome in dialysis patients: Causes and consequences. Am J Kidney Dis.

[19] Forouhi NG, Sattar N, McKeigue PM (2001). Relation of C-reactive protein to body fat distribution and features of the metabolic syndrome in Europeans and South Asians. Int J Obes Relat Metab Disord.

[20] Vikram NK, Misra A, Dwivedi M, Sharma R, Pandey RM, Luthra K (2003). Correlations of C-reactive protein levels with anthropometric profile, percentage of body fat and lipids in healthy adolescents and young adults in urban North India. Atherosclerosis.

[21] Macdougall IC, Cooper AC (2005). Hyporesponsiveness to erythropoietic therapy due to chronic inflammation. Eur J Clin Invest.

[22] Cooper A, Mikhail A, Lethbridge MW, Kemeny DM, Macdougall IC (2004). Pentoxifylline improves hemoglobin levels in patients with erythropoietin-resistant anemia in renal failure. J Am Soc Nephrol.

[23] Kulaksiz H, Gehrke SG, Janetzko A, Rost D, Bruckner T, Kallinowski B (2004). Pro-hepcidin: Expression and cell specific localisation in the liver and its regulation in hereditary haemochromatosis, chronic renal insufficiency, and renal anaemia. Gut.

[24] Park CH, Valore EV, Waring AJ, Ganz T (2001). Hepcidin, a urinary antimicrobial peptide synthesized in the liver. J Biol Chem.

[25] Pietrangelo A, Trautwein C (2004). Mechanisms of disease: The role of hepcidin in iron homeostasis--implications for hemochromatosis and other disorders. Nat Clin Pract Gastroenterol Hepatol.

[26] Malyszko J, Malyszko JS, Hryszko T, Pawlak K, Mysliwiec M (2005). Is hepcidin a link between anemia, inflammation and liver function in hemodialyzed patients?. Am J Nephrol.

[27] Malyszko J, Malyszko JS, Mysliwiec M (2009). Hyporesponsiveness to erythropoietin therapy in hemodialyzed patients: Potential role of prohepcidin, hepcidin, and inflammation. Ren Fail.

[28] Memoli B, Salerno S, Procino A, Postiglione L, Morelli S, Sirico ML (2010). A translational approach to micro-inflammation in end-stage renal disease: molecular effects of low levels of interleukin-6. Clin Sci (Lond).

[29] Hsu SP, Chiang CK, Chien CT, Hung KY (2006). Plasma prohepcidin positively correlates with hematocrit in chronic hemodialysis patients. Blood Purif.

[30] Kato A, Tsuji T, Luo J, Sakao Y, Yasuda H, Hishida A (2008). Association of prohepcidin and hepcidin-25 with erythropoietin response and ferritin in hemodialysis patients. Am J Nephrol.

[31] Shinzato T, Abe K, Furusu A, Harada T, Shinzato K, Miyazaki M (2008). Serum pro-hepcidin level and iron homeostasis in Japanese dialysis patients with erythropoietin (EPO)-resistant anemia. Med Sci Monit.

[32] Zaritsky J, Young B, Wang HJ, Westerman M, Olbina G, Nemeth E (2009). Hepcidin: A potential novel biomarker for iron status in chronic kidney disease. Clin J Am Soc Nephrol.

[33] Deicher R, Horl WH (2004). Hepcidin: A molecular link between inflammation and anaemia. Nephrol Dial Transplant.

[34] Garneata L (2008). Intravenous iron, inflammation, and oxidative stress: Is iron a friend or an enemy of uremic patients?. J Ren Nutr.

[35] Agarwal R (2006). Proinflammatory effects of iron sucrose in chronic kidney disease. Kidney Int.

[36] Agarwal R, Vasavada N, Sachs NG, Chase S (2004). Oxidative stress and renal injury with intravenous iron in patients with chronic kidney disease. Kidney Int.

[37] Anirban G, Kohli HS, Jha V, Gupta KL, Sakhuja V (2008). The comparative safety of various intravenous iron preparations in chronic kidney disease patients. Ren Fail.

[38] Ganguli A, Kohli HS, Khullar M, Lal Gupta K, Jha V, Sakhuja V (2009). Lipid peroxidation products formation with various intravenous iron preparations in chronic kidney disease. Ren Fail.

[39] Maruyama Y, Nakayama M, Yoshimura K, Nakano H, Yamamoto H, Yokoyama K (2007). Effect of repeated intravenous iron administration in haemodialysis patients on serum 8-hydroxy-2’-deoxyguanosine levels. Nephrol Dial Transplant.

[40] Malyszko J, Malyszko JS, Mysliwiec M (2009). Serum prohepcidin and hepcidin in hemodialyzed patients undergoing iron therapy. Kidney Blood Press Res.

[41] Zager RA, Johnson AC, Hanson SY, Lund S (2005). Parenteral iron compounds sensitize mice to injury-initiated TNF-alpha mRNA production and TNF-alpha release. Am J Physiol Renal Physiol.

[42] Weiss G, Meusburger E, Radacher G, Garimorth K, Neyer U, Mayer G (2003). Effect of iron treatment on circulating cytokine levels in ESRD patients receiving recombinant human erythropoietin. Kidney Int.

[43] Slotki I (2005). Intravenous iron supplementation in the anaemia of renal and cardiac failure: A double-edged sword?. Nephrol Dial Transplant.

